# 4-oxo-N-(4-hydroxyphenyl)retinamide: Two Independent Ways to Kill Cancer Cells

**DOI:** 10.1371/journal.pone.0013362

**Published:** 2010-10-14

**Authors:** Paola Tiberio, Elena Cavadini, Gabriella Abolafio, Franca Formelli, Valentina Appierto

**Affiliations:** Department of Experimental Oncology and Molecular Medicine, Fondazione IRCCS “Istituto Nazionale dei Tumori”, Milan, Italy; University of Illinois at Chicago, United States of America

## Abstract

**Background:**

The retinoid 4-oxo-N-(4-hydroxyphenyl)retinamide (4-oxo-4-HPR) is a polar metabolite of fenretinide (4-HPR) very effective in killing cancer cells of different histotypes, able to inhibit 4-HPR-resistant cell growth and to act synergistically in combination with the parent drug. Unlike 4-HPR and other retinoids, 4-oxo-4-HPR inhibits tubulin polymerization, leading to multipolar spindle formation and mitotic arrest. Here we investigated whether 4-oxo-4-HPR, like 4-HPR, triggered cell death also via reactive oxygen species (ROS) generation and whether its antimicrotubule activity was related to a ROS-dependent mechanism in ovarian (A2780), breast (T47D), cervical (HeLa) and neuroblastoma (SK-N-BE) cancer cell lines.

**Methodology/Principal Findings:**

We provided evidence that 4-oxo-4-HPR, besides acting as an antimicrotubule agent, induced apoptosis through a signaling cascade starting from ROS generation and involving endoplasmic reticulum (ER) stress response, Jun N-terminal Kinase (JNK) activation, and upregulation of the proapoptotic PLAcental Bone morphogenetic protein (PLAB). Through time-course analysis and inhibition of the ROS-related signaling pathway (upstream by vitamin C and downstream by PLAB silencing), we demonstrated that the antimitotic activity of 4-oxo-4-HPR was independent from the oxidative stress induced by the retinoid. In fact, ROS generation occurred earlier than mitotic arrest (within 30 minutes and 2 hours, respectively) and abrogation of the ROS-related signaling pathway did not prevent the 4-oxo-4-HPR-induced mitotic arrest.

**Conclusions/Significance:**

These data indicate that 4-oxo-4-HPR anticancer activity is due to at least two independent mechanisms and provide an explanation of the ability of 4-oxo-4-HPR to be more potent than the parent drug and to be effective also in 4-HPR-resistant cell lines. In addition, the double mechanism of action could allow 4-oxo-4-HPR to efficiently target tumour and to eventually counteract the development of drug resistance.

## Introduction

Retinoids are a class of chemical compounds structurally related to vitamin A that modulate fundamental cellular processes, including cell proliferation, differentiation and apoptosis [1]. The synthetic retinoid fenretinide or N-(4-hydroxyphenyl)retinamide (4-HPR) is a non toxic analog of all-trans retinoic acid [2] that has already shown promising results in preneoplastic [3]–[5] and neoplastic conditions [6], [7]. In cultured cells, 4-HPR has been shown to induce growth inhibition and apoptosis in various cancer cell lines and different mechanisms of action have been proposed, including the generation of reactive oxygen species (ROS) and consequent oxidative stress [8], [9]. We have recently reported that in ovarian cancer cells, 4-HPR-induced apoptosis is mediated by the proapoptotic PLAcental Bone morphogenetic protein (PLAB) and that its upregulation by 4-HPR occurs through the activation of a signaling cascade starting from increase of ROS generation, leading to induction of endoplasmic reticulum (ER) stress response and Jun N-terminal Kinase (JNK) activation [9], [10]. From the analysis of plasma samples of 4-HPR-treated patients, we have identified a new 4-HPR polar metabolite, 4-oxo-N-(4-hydroxyphenyl)retinamide (4-oxo-4-HPR) [11], which is endowed with promising biological properties [12]. 4-oxo-4-HPR elicits antiproliferative and apoptotic effects in various cancer cell lines (i.e. ovarian, breast, and neuroblastoma tumour cell lines) and is two to four times more effective than 4-HPR in inhibiting cell growth [12]. Interestingly, 4-oxo-4-HPR is also effective in 4-HPR-resistant cells and, in combination with 4-HPR, has a synergistic effect [12]. Similarly to 4-HPR, the tumour growth-inhibitory effects of 4-oxo-4-HPR are independent of nuclear retinoid receptors (RARs). In addition, 4-HPR and 4-oxo-4-HPR share several signaling intermediates, such as ROS generation, increase of intracellular ceramide levels, and activation of caspase-3 and caspase-9 [12]. Despites these similarities, 4-oxo-4-HPR seems to have additional mechanisms of action compared to the parent drug, also suggested by its ability to be effective in 4-HPR resistant cells [12]. In fact, unlike 4-HPR, 4-oxo-4-HPR causes a marked accumulation of cells in mitotic phase, specifically in pre-anaphase, coupled with activation of the spindle checkpoint [13]. The 4-oxo-4-HPR-induced arrest in mitosis is associated with aberrant spindle formation (i.e. multipolar organization without loss of centrosome integrity), due to the ability of 4-oxo-4-HPR to target microtubules and to inhibit tubulin polymerization through a direct molecular interaction with tubulin [13].The present study was planned to further dissect 4-oxo-4-HPR mechanisms of action underlying its antiproliferative effect, investigating whether the anticancer activity of the retinoid may arise also from its ability to increase ROS generation and whether the antimitotic activity of the retinoid is related to the oxidative stress. We have herein demonstrated that, like 4-HPR, 4-oxo-4-HPR causes increase of ROS generation, followed by induction of ER stress response, activation of JNK and PLAB upregulation and that this signaling cascade is partially involved in the antiproliferative effect of the retinoid. Moreover, the 4-oxo-4-HPR antimitotic effect is functionally independent from the abovementioned apoptotic cascade, thus indicating that 4-oxo-4-HPR antitumor effect is due to at least two independent mechanisms of action.

## Results

### ROS generation participates in 4-oxo-4-HPR-induced apoptosis in A2780 cells

**Figure 1:**
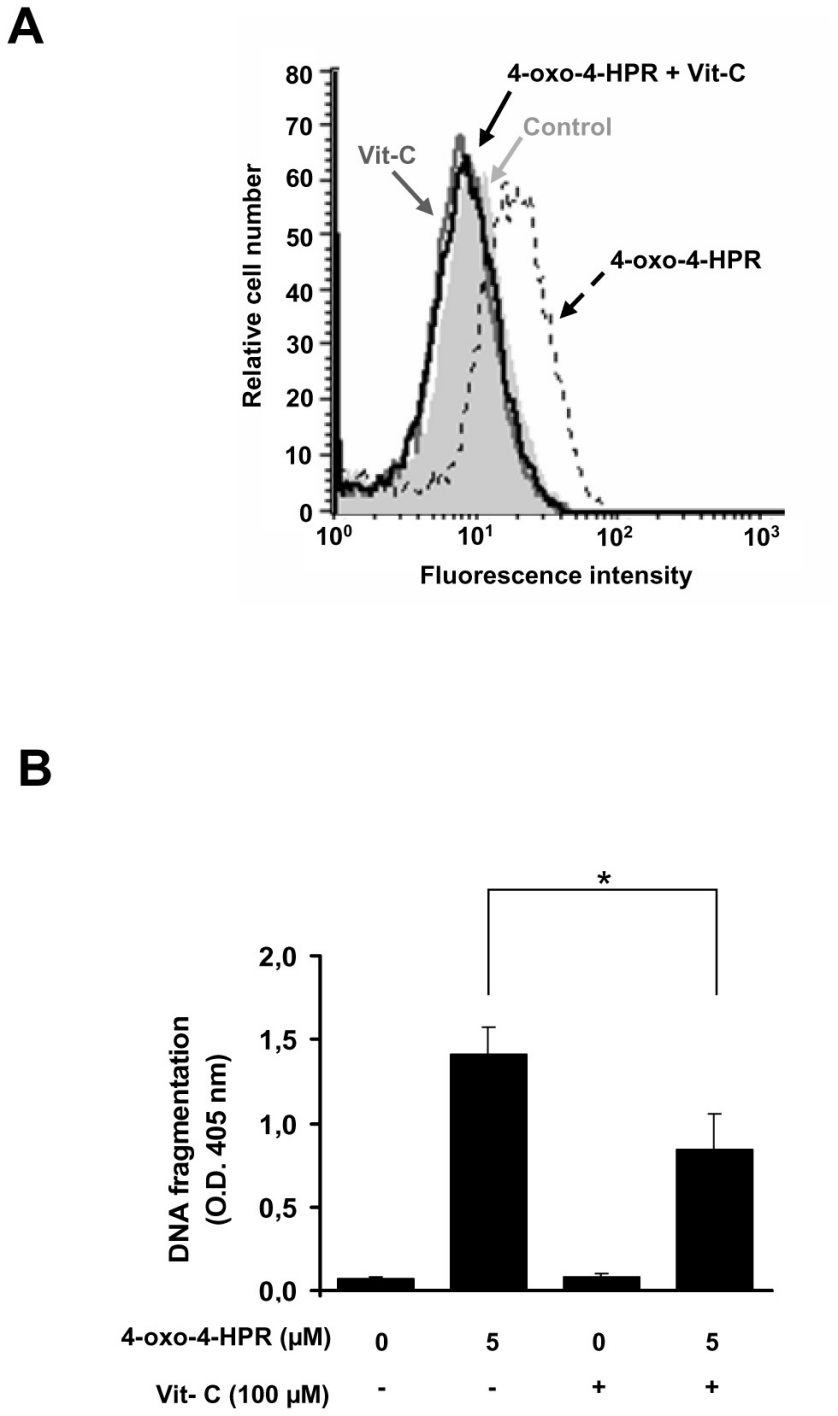
Effects of vitamin C treatment on 4-oxo-4-HPR-induced ROS generation and apoptosis. (A) Analysis of ROS production in A2780 cells treated for 4 hours with 5 µM 4-oxo-4-HPR, with or without 100 µM vitamin C. The analysis was performed by flow cytometry after addition of the redox-sensitive dye CM-H_2_DCFDA. The graph shows representative flow cytometry fluorescence profiles in different conditions of treatment (one representative experiment of three). A shift to the right from control indicates increased ROS levels. (B) A2780 cells were treated for 24 hours with 5 µM 4-oxo-4-HPR, with or without 100 µM vitamin C and apoptosis, evaluated as DNA fragmentation, was measured by an ELISA assay. Data are means of three independent experiments; vertical bars are standard deviations. Asterisk indicates significant difference (P<0.05).

We have recently reported that 4-HPR triggers apoptosis through activation of a signaling cascade that starts from ROS generation and that involves ER stress responses, JNK activation and PLAB upregulation [9]. To investigate if the signaling cascade responsible for 4-HPR-induced apoptosis was also involved in the apoptosis induced by 4-oxo-4-HPR, we first analyzed the involvement of ROS generation in the apoptosis induced by 4-oxo-4-HPR in A2780, a human ovarian carcinoma cell line, chosen because it is already known to be responsive to the retinoid (IC_50_  = 0.6 µM in a 72 hours assay) and to generate ROS in response to 4-oxo-4-HPR treatment [12]. The involvement of ROS production was assayed by evaluating the effect of the antioxidant vitamin C on 4-oxo-4-HPR-induced apoptosis. Five µM 4-oxo-4-HPR treatment for 4 hours caused an increase of ROS production, that was prevented by the addition of 100 µM vitamin C (Figure 1A). Abrogation of ROS generation by vitamin C caused a reduction (1.7 fold) on 4-oxo-4-HPR-induced apoptosis, evaluated as DNA fragmentation by an Elisa assay (Figure 1B). A similar apoptosis reduction has been observed through the determination of sub-G_1_ population by propidium iodide staining followed by flow cytometry analysis (see Figure 5 and relative result paragraph). These data suggested that ROS generation induced by 4-oxo-4-HPR was involved in 4-oxo-4-HPR-induced apoptosis.

### 4-oxo-4-HPR induces ER stress response in A2780 cells

**Figure 2:**
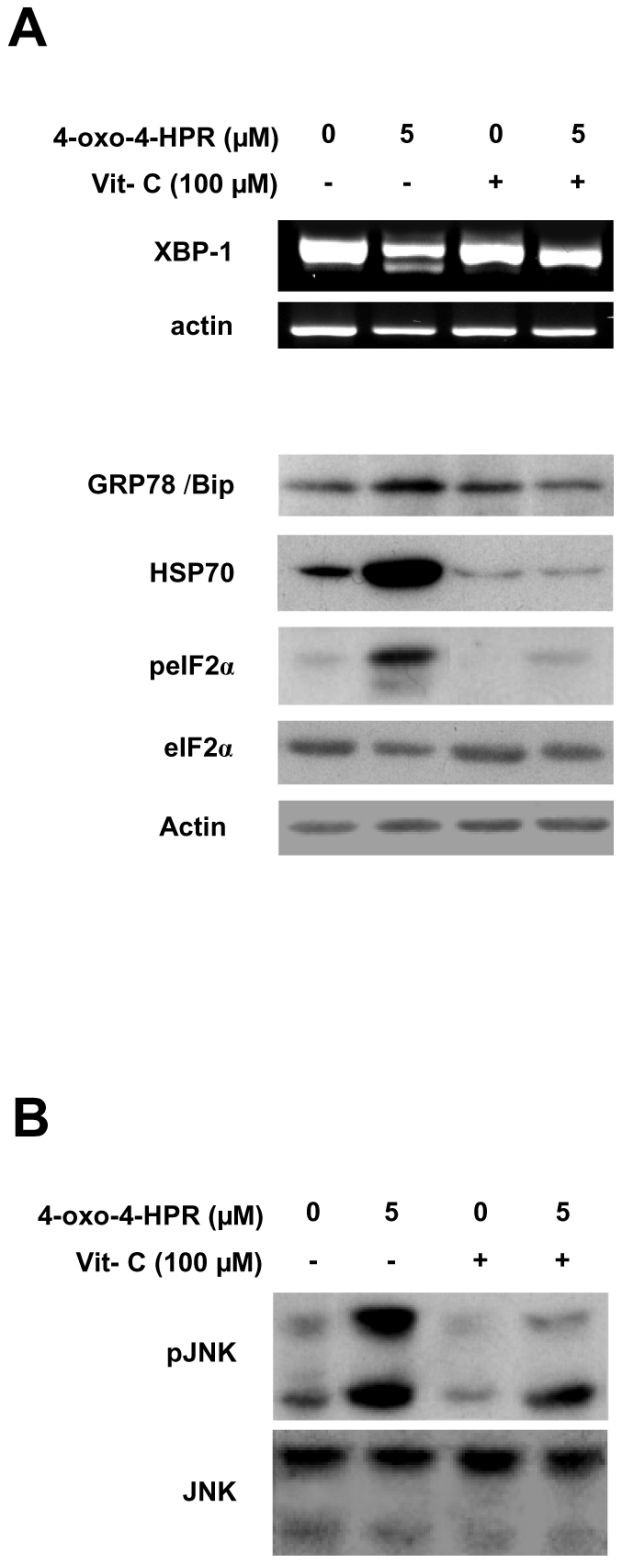
Effects of 4-oxo-4-HPR and vitamin C treatments on ER stress marker expression and JNK phosphorylation. (A) A2780 cells treated for 24 hours with 5 µM 4-oxo-4-HPR, with or without 100 µM vitamin C, were subjected to RT-PCR assay to analyze the splicing of the 25 bp intron from XBP-1 transcript and western blot analysis for the expression of GRP-78/Bip, peIF2α, eIF2α. For reverse transcription–PCR, β-actin was amplified as internal control. For western blot analysis, as a control for loading, the blot was incubated with actin antibody. (B) Cells treated as in (A) were subjected to western blot analysis for the expression of pJNK and JNK.

We analyzed whether 4-oxo-4-HPR, like 4-HPR [9], activated, downstream of ROS generation, an ER stress response, by evaluating ER-stress specific signals: the post-transcriptional splicing of the transcription factor X-box binding protein-1 (XBP-1), the expression of the chaperon proteins glucose-regulated protein 78 KD (GRP-78)/immunoglobulin-binding protein (Bip) and the heat shock protein 70 (HSP70), and the phosphorylation status of the alpha-subunit of eukaryotic initiation factor 2 (eIF2α) [14]. In A2780 cells, 5 µM 4-oxo-4-HPR treatment for 24 hours induced the splicing of a 25 bp intron from the XBP-1 precursor mRNA, caused upregulation of GRP78/Bip and HSP70 and phosphorylation of eIF2α (Figure 2A). The activation of these ER stress-associated events was abrogated (XBP-1, GRP78/Bip and HSP70) or strongly reduced (eIF2α) by the addition of vitamin C (Figure 2A). The results indicated that 4-oxo-4-HPR caused induction of ER stress response, as downstream event of ROS generation.

### 4-oxo-4-HPR induces JNK activation in A2780 cells

We analyzed whether 4-oxo-4-HPR, like 4-HPR [9], induced JNK activation. Western blot analysis, in A2780 cells, showed that 5 µM 4-oxo-4-HPR treatment for 24 hours caused JNK phosphorylation and that the addition of vitamin C strongly reduced the activation of the kinase (Figure 2B). The data indicated that 4-oxo-4-HPR induced the activation of JNK and that this event occurred through a ROS-dependent mechanism.

### PLAB upregulation is functionally involved in apoptosis induced by 4-oxo-4-HPR

**Figure 3:**
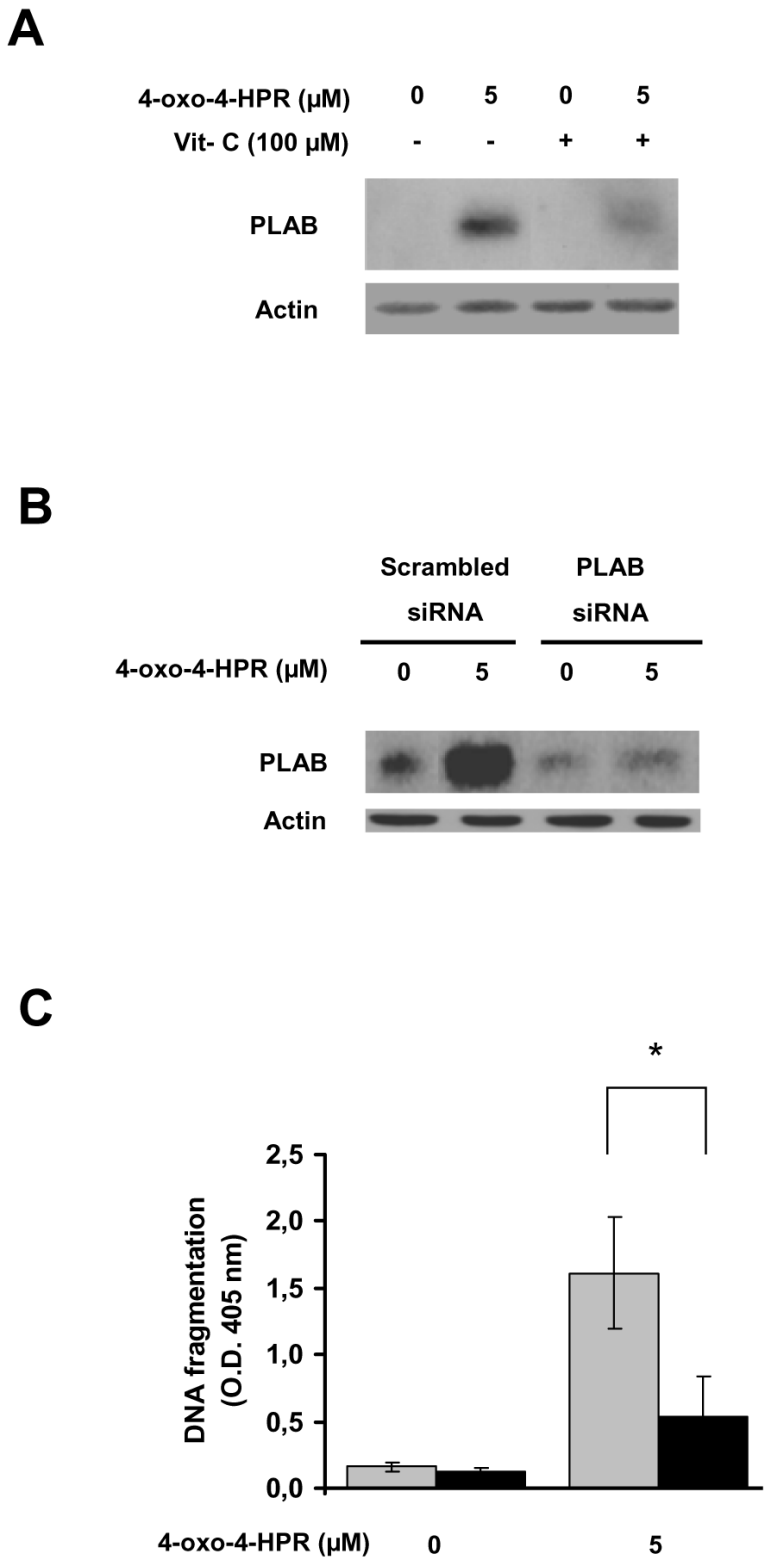
Role of PLAB upmodulation in 4-oxo-4-HPR-induced apoptosis. (A) Western blot analysis for PLAB expression in A2780 cells treated for 24 hours with 5 µM 4-oxo-4-HPR, with or without 100 µM vitamin C. As a control for loading, the blot was incubated with actin antibody. (B) Western blot analysis for PLAB expression in A2780 cells stably transfected with a plasmid containing a PLAB siRNA or a scrambled nonsilencing siRNA following addition of 5 µM 4-oxo-4-HPR for 24 hours. As a control for loading, the blot was incubated with actin antibody. (C) Detection of 4-oxo-4-HPR-induced apoptosis in A2780 stably transfected with a plasmid containing a PLAB siRNA (black columns) or a scrambled nonsilencing siRNA (grey columns). Transfected cells were treated for 24 hours with 5 µM 4-oxo-4-HPR and apoptosis, evaluated as DNA fragmentation, was measured by an ELISA assay. Data are means of four independent experiments; vertical bars are standard deviations. Asterisk indicates significant difference (P<0.01).

We have previously reported that PLAB is upregulated by 4-HPR in a ROS dependent manner [9], and that it plays a functional role in apoptosis induced by the retinoid in A2780 cells [10]. We thus evaluated whether also 4-oxo-4-HPR modulated PLAB expression. Western blot analysis showed that 5 µM 4-oxo-4-HPR treatment for 24 hours induced PLAB upregulation and that this effect was strongly reduced by the addition of vitamin C (Figure 3A). To investigate whether PLAB upregulation played a functional role in the apoptosis induced by 4-oxo-4-HPR, we took advantage of PLAB silencing in A2780 cells, previously generated by stable transfection [10]. As expected, PLAB upmodulation induced by 4-oxo-4-HPR was strongly reduced in cells transfected with the PLAB siRNA plasmid compared with cells transfected with a scrambled plasmid used as control (Figure 3B). As shown in Figure 3C, the apoptosis induced by 4-oxo-4-HPR in cells transfected with PLAB siRNA was approximately 2-fold reduced versus the cells transfected with the scrambled plasmid. The reduction of apoptosis induced by 4-oxo-4-HPR in PLAB silenced cells has also been confirmed by evaluation of sub-G_1_ population after propidium iodide staining (data not shown). The results demonstrated that 4-oxo-4-HPR induced PLAB upregulation as an event downstream of ROS generation and that PLAB contributed to 4-oxo-4-HPR-induced apoptosis. All the aforementioned data suggested that 4-oxo-4-HPR caused the activation of the ROS-related signalling cascade already shown to be activated by 4-HPR (ROS → ER stress → JNK → PLAB) [9].

### 4-oxo-4-HPR induces Bcl-2 and Mcl-1 downregulation in a ROS-independent manner

Since it has been reported that 4-HPR can induce apoptosis through regulation of members of the Bcl-2 family, as downstream event of ROS generation [15]–[17], we analyzed the effect of 4-oxo-4-HPR on the antiapoptotic proteins B cell lymphoma gene-2 (Bcl-2) and myeloid cell leukemia-1 (Mcl-1) and on the proapoptotic protein Bcl-2-associated X protein (Bax). In A2780 cells, 5 µM 4-oxo-4-HPR treatment for 24 hours caused a decrease of Bcl-2 and Mcl-1 protein expression level, whereas had not effect on Bax expression (Figure S1). The downregulation of both Bcl-2 and Mcl-1 was not prevented by the addition of vitamin C (Figure S1), thus suggesting that 4-oxo-4-HPR caused such a decrease in a ROS-independent manner.

### Mitotic arrest and formation of multipolar spindles are independent from ROS-related signaling cascade in A2780 cells

**Figure 4:**
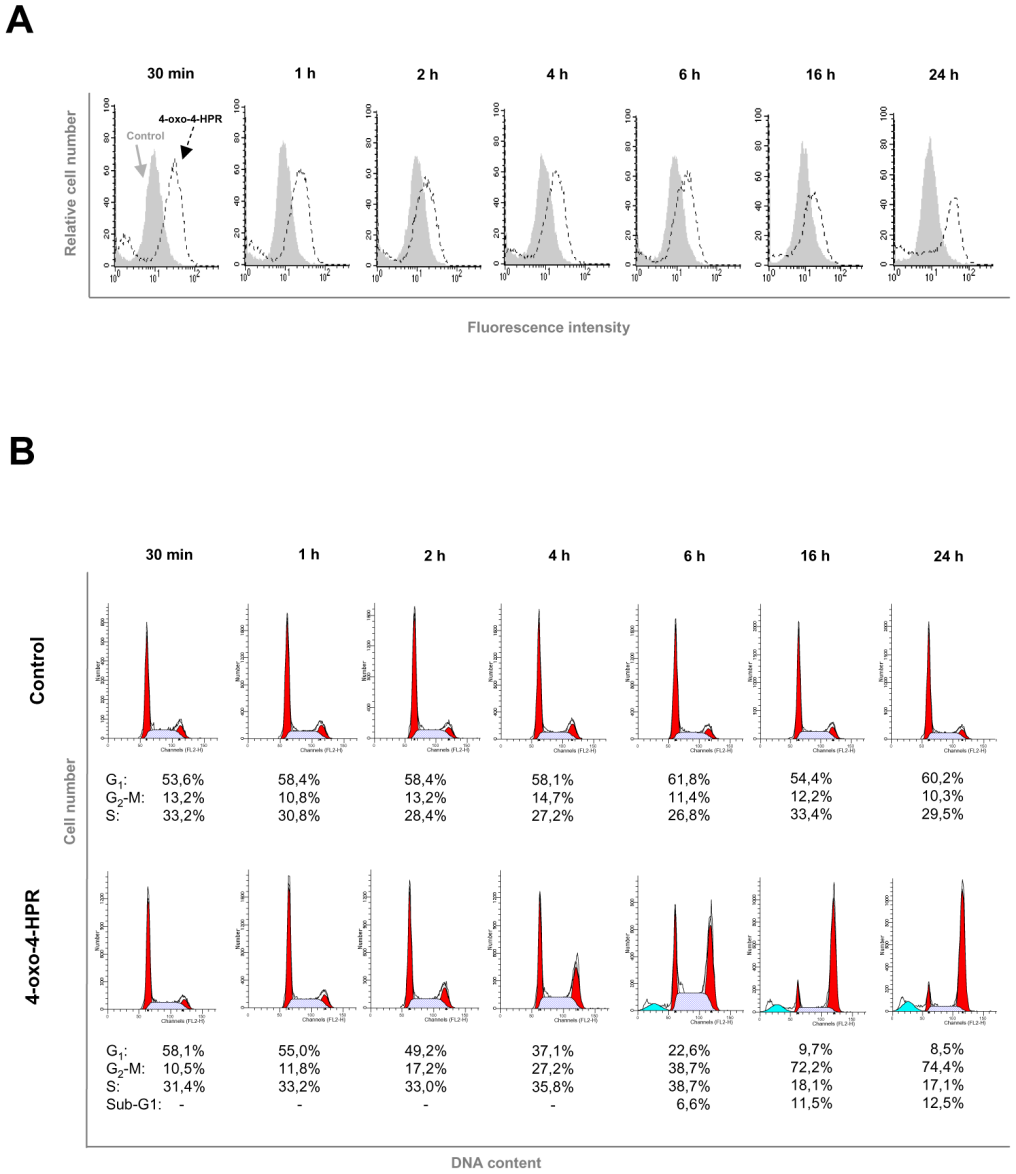
Effects of 4-oxo-4-HPR treatment at different time points on ROS generation and cell cycle distribution. (A) Analysis of ROS production in A2780 cells treated with 5 µM 4-oxo-4-HPR for 30 minutes, 1 hour, 2 hours, 4 hours, 6 hours, 16 hours, and 24 hours. The analysis was performed by flow cytometry after addition of the redox-sensitive dye CM-H_2_DCFDA. The graphs show representative flow cytometry fluorescence profiles in different conditions of treatment (one representative experiment of three). A shift to the right from control indicates increased ROS levels. (B) Flow cytometric analysis of propidium iodide-stained A2780 cells treated with vehicle or 5 µM 4-oxo-4-HPR for 30 minutes, 1 hour, 2 hours, 4 hours, 6 hours, 16 hours, and 24 hours. Numbers in the figure indicate the percentage of cells in the phase of cell cycle, according to the analysis performed with ModFit LT software. One experiment representative of three is shown.

**Figure 5:**
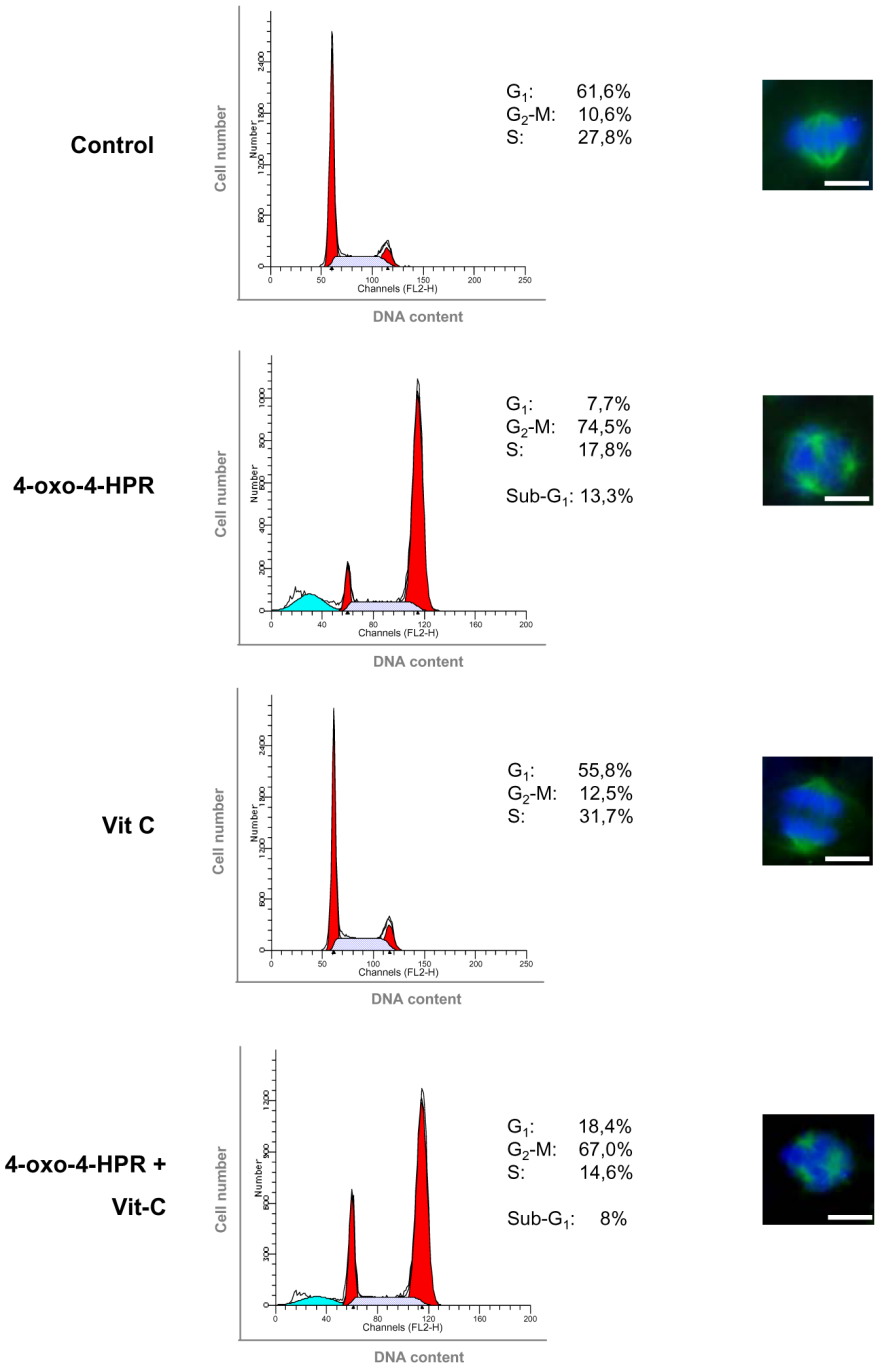
Effects of 4-oxo-4-HPR and vitamin C treatments on cell cycle distribution and spindle assembly. Flow cytometric analysis of propidium iodide-stained A2780 cells treated for 24 hours with 5 µM 4-oxo-4-HPR with or without 100 µM vitamin C. Numbers in the figure indicate the percentage of cells in the phase of cell cycle, according to the analysis performed with ModFit LT software. One experiment representative of three is shown. On the right is depicted a representative mitotic cell image for each treatment, obtained by immunostaining with α-tubulin antibody (*green*) and nuclear staining with Hoechst 33342 (*blue*). Scale bar  = 5 µm.

Contrary to 4-HPR, which only slightly affects the G_1_ phase of the cell cycle, 4-oxo-4-HPR causes a marked accumulation of cells in G_2_-M phases [12]. We have recently reported that 4-oxo-4-HPR-induced cell cycle perturbation was due to the ability of the retinoid to inhibit tubulin polymerization, arresting cells in mitosis [13]. Moreover, the antimitotic effect of the retinoid was shown to be coupled with formation of aberrantly shaped spindles (i.e. multipolars) due to its effects on tubulin polymerization [13]. To investigate whether the 4-oxo-4-HPR-induced mitotic arrest and the formation of multipolar spindles were related to the ROS-induced signaling cascade we first performed a time-course analysis of both ROS generation and cell cycle perturbation induced by 4-oxo-4-HPR. ROS generation was observed as early as 30 minutes after retinoid treatment (Figure 4A) and stayed throughout the 24-hour time-course. By contrast, a slight G_2_-M cell accumulation was observed only at 2 hours and the increase of the percentage of G_2_-M arrested cells was time-dependent up to 16-24 hours (Figure 4B). The time-course analysis thus revealed that the oxidative stress induced by 4-oxo-4-HPR occurred earlier than the cell cycle arrest and that the two events presented different kinetics. To investigate whether the 4-oxo-4-HPR-induced ROS generation played a role in the mitotic arrest, we analyzed the effects of vitamin C on cell cycle perturbation and formation of aberrant spindles induced by the retinoid. In A2780 cells exposed for 24 hours to 5 µM 4-oxo-4-HPR, the addition of 100 µM vitamin C did not prevent the accumulation of G_2_-M cell population (Figure 5 and S2) or the formation of multipolar spindles (Figure 5). Consistent results in cell cycle distribution and formation of multipolar spindles were obtained by inhibiting the ROS-related signaling cascade at a downstream level through PLAB silencing: transfection with PLAB siRNA did not prevent 4-oxo-4-HPR-induced mitotic arrest and formation of multipolar spindles (data not shown). The results indicated that in A2780 cells, the mitotic arrest induced by 4-oxo-4-HPR was neither an event downstream of ROS generation, nor a step of the ROS-related signaling cascade.

### 4-oxo-4-HPR acts through a double mechanism of action also in cancer cell lines of different histotypes

**Figure 6:**
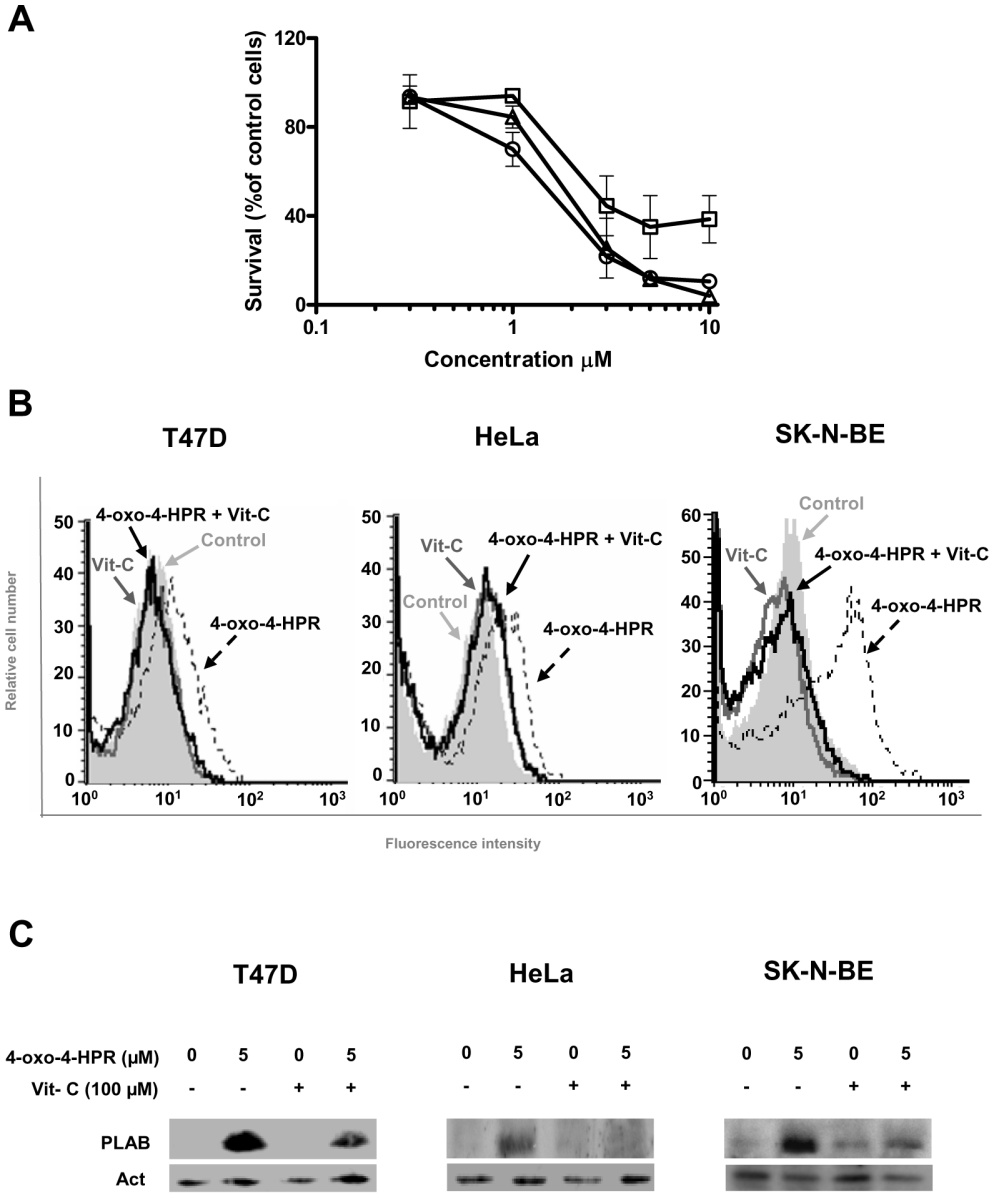
Effects of 4-oxo-4-HPR and vitamin C treatments on ROS generation and PLAB expression. (A) Citotoxic effect of 4-oxo-4-HPR on T47D (□), HeLa (▵) and SK-N-BE (○) cell growth was estimated by using the sulforhodamine B assay. The antiproliferative activity of 4-oxo-4-HPR in each cell line was tested in three independent experiments with four replicate wells for each analysis; vertical bars are standard deviations. (B) Analysis of ROS production in T47D, HeLa and SK-N-BE cells treated for 4 hours with 5 µM 4-oxo-4-HPR, with or without 100 µM vitamin C. The analysis was performed by flow cytometry after addition of the redox-sensitive dye CM-H_2_DCFDA. The graphs show representative flow cytometry fluorescence profiles in different conditions of treatment (one representative experiment of three). A shift to the right from control indicates increased ROS levels. (C) Western blot analyses for PLAB expression in T47D, HeLa and SK-N-BE cells treated with 5 µM 4-oxo-4-HPR for 24 hours, with or without 100 µM vitamin C. As a control for loading, the blot was incubated with actin antibody.

**Figure 7:**
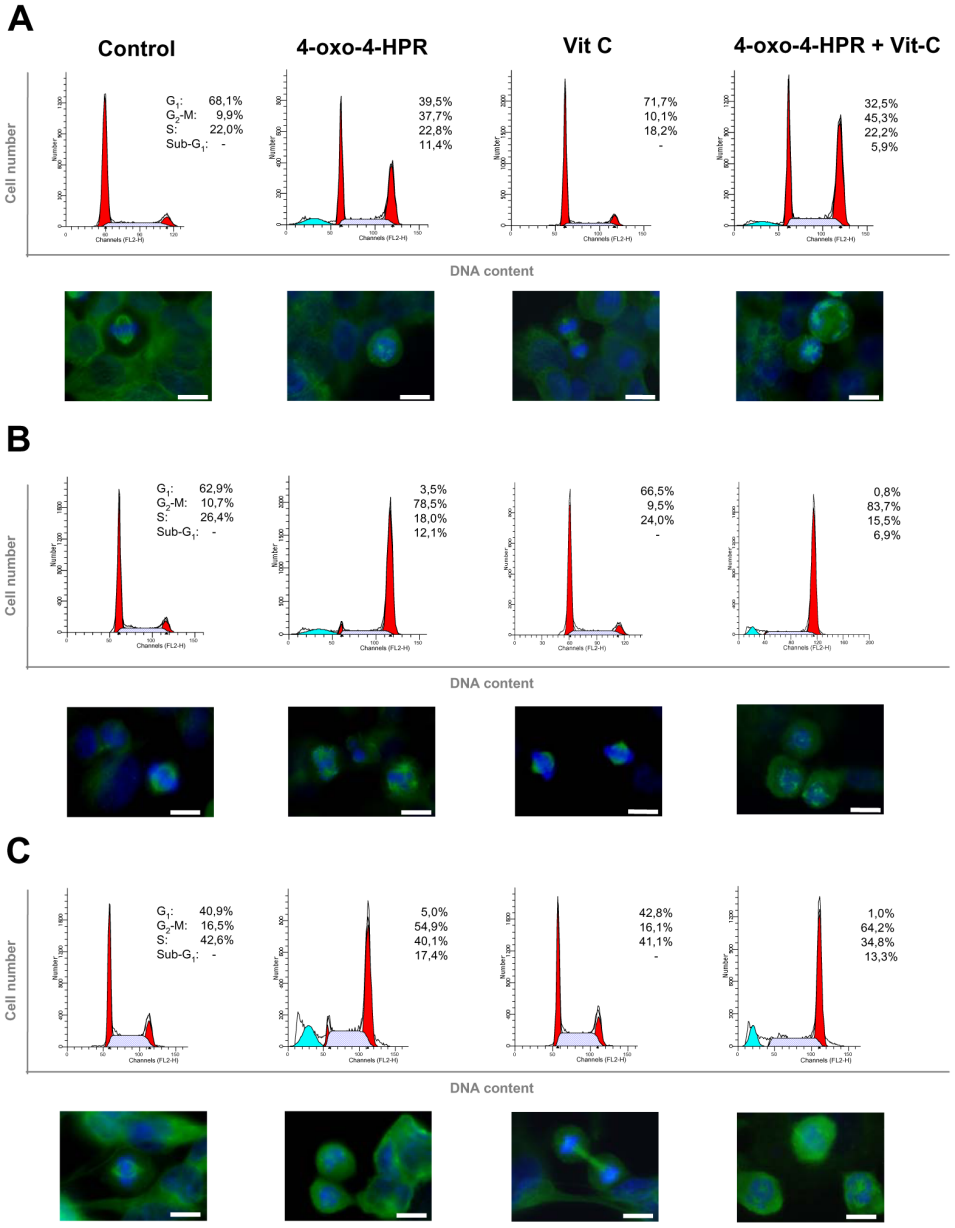
Effects of vitamin C treatment on 4-oxo-4-HPR antimitotic activities in T47D, HeLa and SK-N-BE cells. Flow cytometric analysis of propidium iodide-stained T47D (A), HeLa (B) and SK-N-BE (C) cells treated for 24 hours with 5 µM 4-oxo-4-HPR with or without 100 µM vitamin C. Numbers in the figure indicate the percentage of cells in the phase of cell cycle, according to the analysis performed with ModFit LT software. One experiment representative of three is shown. On the bottom of each histogram, immunofluorescence analysis with α-tubulin antibody (*green*) of cells treated in the same way. Nuclear morphology was visualized by staining with Hoechst 33342 (*blue*). Scale bar  = 10 µm.

To determine whether the involvement of the two independent mechanisms in 4-oxo-4-HPR activity was restricted to A2780 cells or represented the distinctive mode of action of the retinoid, we extended the analysis of 4-oxo-4-HPR effects to three others human cancer cell lines responsive to the retinoid: T47D (mammary adenocarcinoma), HeLa (epithelial cervical adenocarcinoma) and SK-N-BE (neuroblastoma) cells (Figure 6A). We first assessed the intracellular generation of ROS induced by 4-oxo-4-HPR showing that, in the three cancer cell lines, 5 µM 4-oxo-4-HPR for 4 hours increased ROS production over controls and that the addition of 100 µM vitamin C inhibited the ROS generation induced by the retinoid (Figure 6B). In the three tested cell lines, 4-oxo-4-HPR treatment induced PLAB upregulation and this effect was reduced by the addition of vitamin C (Figure 6C). We then performed cell cycle analysis showing that 4-oxo-4-HPR treatment induced a marked G_2_-M cell cycle arrest in all tested cancer cell lines (Figure 7 and S2). Similarly to what observed in A2780 cells, in these cell lines the inhibition of ROS generation with vitamin C caused a marked reduction of sub-G_1_ population, but did not decrease the percentage of cells arrested in G_2_-M phase (Figure 7 and S2). Moreover, 4-oxo-4-HPR treatment caused formation of multipolar spindles in T47D, HeLa and SK-N-BE cells (Figure 7), and in all of them the addition of vitamin C did not prevent the formation of aberrant spindles induced by the retinoid (Figure 7). The results suggested that, also in T47D, HeLa and SK-N-BE cells, the ROS-dependent signaling cascade was involved in 4-oxo-4-HPR-induced apoptosis. Moreover, 4-oxo-4-HPR-induced cell cycle arrest and the formation of multipolar spindles were independent from the ROS-associated signaling cascade, not only in ovarian but also in breast, cervical and neuroblastoma cancer cell lines, revealing the distinctive feature of 4-oxo-4-HPR to have a double mechanism of action.

## Discussion

4-oxo-4-HPR is a polar metabolite of the synthetic retinoid 4-HPR that was detected in plasma samples of women treated with 4-HPR participating in a Phase III breast cancer prevention trial [11]. Our previous *in vitro* studies conducted with 4-oxo-4-HPR have shown that the retinoid is endowed with very promising anticancer properties, such as higher tumour growth inhibitory effects than 4-HPR (being its IC_50_ values two to four times lower than the parent drug in the majority of tested cell lines), lack of cross resistance and synergistic interaction with the parent drug [12], suggesting that it might be proposed as a new agent for cancer therapy. Differently from 4-HPR and other retinoids, 4-oxo-4-HPR targets microtubules and inhibits tubulin polymerization causing mitotic arrest and formation of multipolar spindles without loss of centrosome integrity [13]. On the other hand, similarly to the parent drug, 4-oxo-4-HPR induces increase of ROS generation [12].

Since the 4-HPR-induced ROS generation has been shown to activate an apoptotic cascade involving ER stress response, JNK activation and upregulation of the proapoptotic protein PLAB [9], we decided to investigate whether 4-oxo-4-HPR triggered apoptosis also via ROS generation and whether its antimitotic activity was related to this ROS-dependent pathway. 4-oxo-4-HPR-induced ROS production contributed to the apoptotic activity of the retinoid, because the treatment with the antioxidant vitamin C caused a reduction of 4-oxo-4-HPR-induced apoptosis. In our previous study on 4-oxo-4-HPR characterization [12], we were not able to show a clear causal relationship between generation of ROS and cell growth inhibitory activity of the retinoid. A possible explanation could be that the antioxidant used in the previous analysis (i.e. *N*-acetyl-L-cysteine) did not totally prevent the oxidative stress induced by 4-oxo-4-HPR, but only caused a partial reduction of ROS production [12].

Downstream of ROS generation, 4-oxo-4-HPR activated other signaling intermediates of the 4-HPR apoptotic cascade, such as ER stress response (detected by XBP-1 splicing, GRP78/Bip and HSP70 upregulation, and eIF2α phosporylation) and JNK phosporylation, both prevented by vitamin C addition. The expression of the proapoptotic protein PLAB was dramatically increased by 4-oxo-4-HPR and its upregulation was reduced by vitamin C addition, suggesting that the protein expression modulation was a downstream event of the oxidative stress induced by the retinoid. Moreover, we demonstrated that PLAB played a functional role in 4-oxo-4-HPR apoptotic activity, because its silencing decreased the apoptosis induced by the retinoid. Therefore, our results indicated that 4-oxo-4-HPR, besides acting as an antimicrotubule agent, induced apoptosis via the signaling cascade that we have already shown to be activated by 4-HPR (ROS → ER stress → JNK → PLAB) [9].

PLAB apoptotic-inducing activity has been observed and reported by other authors in several cellular contexts and following treatment with different anticancer agents [9], [18]-[21]. However, the specific downstream events by which PLAB mediates such effect remain to be determined. From our results, an involvement of the proteins Bcl-2 and Mcl-1, a Bcl-2 family member, in PLAB apoptotic activity might be possibly excluded. In fact, even though 4-oxo-4-HPR treatment caused a downregulation of the expression level of the two antiapoptotic proteins, such a decrease was not prevented by the inhibition of the ROS-mediated apoptotic cascade involving PLAB.

It is interesting to note that 4-oxo-4-HPR is an oxidized form of 4-HPR and that the modification in position 4 of the cyclohexene ring is probably responsible for 4-oxo-4-HPR antimicrotubule activity, but does not affect the ability of the retinoid to induce ROS generation and to activate the ROS-related signalling cascade. Such chemical modification could be also responsible for the different sphingolipid metabolism observed between the two retinoids. Both 4-HPR and 4-oxo-4-HPR have been shown to induce a dramatic increase of dihydroceramide production; however, the contribution of distinct molecular species to the total increment was reported to be markedly different, in terms of sphingosine/sphinganine and fatty acid content. In addition, 4-oxo-4-HPR, contrary from the parent drug, has been shown to slightly increase also the ceramide production [22].

We have previously reported that 4-oxo-4-HPR acts atypically compared to 4-HPR and other retinoids, due to its ability to inhibit tubulin polymerization, leading to formation of multipolar spindles and mitotic arrest [13]. In this study we have found that the mitotic arrest and the coupled formation of multipolar spindles induced by 4-oxo-4-HPR were independent from the ROS-related signaling cascade. In fact, we have shown that 4-oxo-4-HPR-induced ROS generation occurred earlier than the mitotic arrest, thus indicating that the antimitotic activity of the retinoid was not an event upstream the oxidative stress. On the other hand, the inhibition of the ROS-related pathway by vitamin C or by PLAB silencing did not prevent the ability of 4-oxo-4-HPR to exert its antimicrotubule activity, thus suggesting that the mitotic arrest was not a ROS-dependent mechanism. The occurrence of a double mechanism of action could plausibly be a distinctive feature of the mode of action of 4-oxo-4-HPR, since these two unrelated pathways were found in cancer cell lines of different histotypes (ovarian, breast, cervical carcinoma and neuroblastoma).

**Figure 8:**
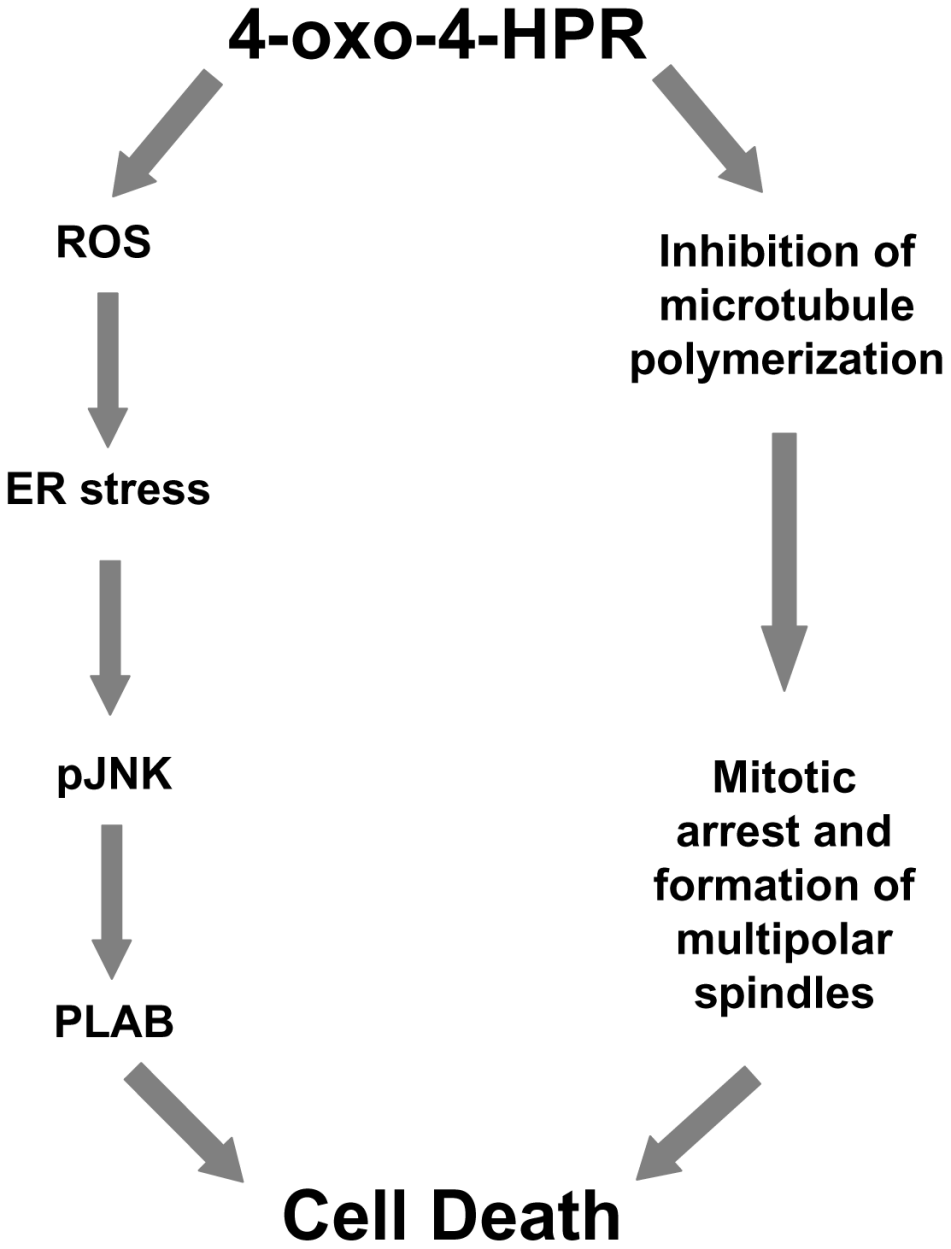
Scheme showing proposed cascade of events involved in 4-oxo-4-HPR-induced growth inhibitory effect. 4-oxo-4-HPR induces cell death through two independent mechanisms of action: 1) a ROS-related signaling cascade involving ER stress response, JNK activation and upregulation of the proapoptotic protein PLAB; and 2) antimicrotubule activities consisting in inhibition of tubulin polymerization, mitotic arrest and formation of multipolar spindles.

Even though the exact mechanism by which 4-oxo-4-HPR antimicrotubule activity triggers cell death has yet to be determined, on the basis of the abovementioned data, we can speculate that both the ROS-related signaling cascade and the antimicrotubule activities independently contribute to 4-oxo-4-HPR antiproliferative effect and we propose that the retinoid acts as presented schematically in Figure 8. Nonetheless, the analysis of 4-oxo-4-HPR mechanisms of action will need further studies to investigate how the two pathways molecularly lead to cell death. It is tempting to speculate that the ROS-independent downregulation of Bcl-2 and Mcl-1, observed after 4-oxo-4-HPR treatment, could play a role in the apoptosis induced by the antimicrotubule activity of the retinoid, although at present no evidence supports this hypothesis. According to our observations, it has been reported that treatment with other microtubule-interfering agents can lead to a decrease in Bcl-2 and Mcl-1 intracellular amounts contributing to apoptosis [23]–[25].

It is noteworthy that other microtubule-targeted agents have been shown to promote ROS generation in cancer cells, but the relationship between tubulin dynamics and oxidative stress remain unclear [26]–[33]. Recently, the anticancer activity of paclitaxel has been linked to the generation of intracellular and extracellular ROS and it has been suggested that the disturbance of microtubule polymerization state induced by this agent, as well as by taxotere or vincristine, could enhance ROS production through stabilization of active NADPH oxidase [31], [32]. ROS generation contributed also to the antiproliferative effect of patupilone, a member of the microtubule-stabilizing agents epothilones, and a causal relationship between the oxidative stress and the modifications of microtubule dynamics has been suggested [30]. By contrast, the ROS generation induced by stilbene 5c, a microtubule inhibitor at the colchicine site, has been shown to be unrelated to the drug-induced cell cycle perturbation [29], similarly to what we have found for 4-oxo-4-HPR.

The ability of 4-oxo-4-HPR to act through at least two unrelated mechanisms could probably allow to counteract the development of drug resistance. In fact, we have shown that if one pathway is inhibited (i.e. ROS-related signaling pathway), the retinoid is able to act through the other mechanism (i.e. mitotic arrest) to kill cancer cells. In addition, the knowledge of the mechanisms underlying 4-oxo-4-HPR antiproliferative activity might facilitate future design of drug combination strategies.

In conclusion, the new information provided by our study on the mechanism of action of 4-oxo-4-HPR is that the retinoid exerts its activity through at least two independent pathways that contribute to its antiproliferative effect, i.e. the ROS-related signaling cascade and the antimicrotubule activity. The finding of a double mechanism underlying anticancer activities of 4-oxo-4-HPR provides an explanation of the ability of 4-oxo-4-HPR to be more potent than the parent drug and to be effective also in 4-HPR-resistant cell lines (i.e. A2780/HPR cells) [12]. This distinctive mode of action could allow 4-oxo-4-HPR to efficiently target tumor and eventually to counteract the development of drug resistance.

## Materials and Methods

### Cell lines and reagents

Ovarian tumour cell line A2780 (obtained from Dr. Ozols, Bethesda, MD) and neuroblastoma cell line SK-N-BE, purchased from ATCC (Manassas, VA, USA), were maintained in RPMI 1640 (Lonza, Basel, Switzerland) containing 10% fetal calf serum. Breast tumour cell line T47-D (obtained from Dr. R. Sutherland, Sydney, New South Wales, Australia) was maintained in RPMI 1640 containing 10% fetal calf serum and 0.25 U/mL insulin. Cervical carcinoma cell line HeLa, purchased from ATCC, was manteined in Dulbecco's Modified Eagle medium (Gibco Brl, Paisley, UK) supplemented with 10% fetal bovine serum. Stably transfected A2780 cells with PLAB siRNA or a scrambled nonsilencing siRNA were generated previously [10] and maintained in RPMI 1640 medium supplemented with 10% fetal calf serum and containing G418 (Gibco) at a concentration of 400 mg/ml. All cell lines were cultured at 37°C under 5% CO_2_. 4-oxo-4-HPR, synthesized as previously described [11], was dissolved at 10 mmol/L in DMSO prior to further dilution in culture medium and stored at –80°C in the dark. Vitamin C (Sigma, St Louis, MO, USA) was added to cells 1 h before 4-oxo-4-HPR treatment.

### Growth inhibition assay

T47D, HeLa and SK-N-BE cells were seeded in 96-well tissue culture plates at 7×10^3^ cells/well and were allowed to adhere for 24 hours before treatment. Cells were grown in the presence of vehicle or 4-oxo-4-HPR at a final concentration of 0.3, 1, 3, 5, and 10 µM (4 wells for each treatment). Cellular growth was assessed after 72 hours by sulforhodamine B (SRB) assay. Briefly, proteins were precipitated with 10% trichloroacetic acid for 1 hour at 4°C and stained for 30 minutes with SRB dye 0.4% w/v in acetic acid 1% v/v. Finally, precipitated proteins were washed and solubilized in Tris buffer 10 mM. Absorbance [optical density (OD)] was measured at 540 nm by using a microplate reader. For each treatment, cell survival was estimated from the equation: % cell survival  = 100× At/Ac, where At and Ac are the absorbencies of the sulforhodamine B color reaction in treated and control cultures, respectively. The antiproliferative activity of 4-oxo-4-HPR in each cell line was tested in three independent experiments with four replicate wells for each analysis.

### Determination of reactive oxygen species

Intracellular production of ROS was detected by using of the oxidation-sensitive dye 5-(and-6)-chloromethyl-2′,7′-dichlorodihydrofluorescein diacetate (CM-H_2_DCFDA; Molecular Probes, Inc., Eugene, OR) as described previously [12]. Briefly, cells (8×10^5^ per well) were plated in six-well cell culture plates and incubated for different time points in the presence of 5 µM 4-oxo-4-HPR. Medium was discarded and, under low light conditions, replaced with 50 µM CM-H_2_DCFDA in whole medium for 20 minutes at 37°C. Cells were harvested, transferred to foil-wrapped tubes and analyzed immediately by flow cytometry.

### Immunoblot analysis

Proteins were extracted by lysing cells in sodium dodecyl sulfate (SDS) sample buffer (62.5 mM Tris–HCl [pH 6.8], 2% SDS) containing 1 mM phenylmethylsulfonyl fluoride, 10 µg/mL pepstatin, 12.5 µg/mL leupeptin, 2 µg/mL aprotinin, 1 mM sodium orthovanadate, and 1 mM sodium molybdate. Cell extracts were processed for western immunoblotting as described previously [34]. The following antibodies used for immunoblotting were purchased from the indicated suppliers: PLAB and GRP-78/Bip from Santa Cruz Biotechnology (Santa Cruz, CA, USA); HSP70, phospho-JNK (Thr183/Tyr185), JNK1/2, phospho-eIF2α, eIF2α and Mcl-1 from Cell Signaling Biotechnology (Beverly, MA, USA), Bcl-2 from DakoCytomation (Glostrup, Denmark), and Bax and actin from Sigma.

### Apoptosis evaluation

Fragmentation of DNA was determined by photometric enzyme immunoassay using Cell Death Detection ELISAplus kit (Roche, Penzberg, Germany) according to the manufacturer's instruction. Briefly, cells (1×10^4^ per well) were seeded in 96-well cell culture plates and treated the day after. Adherent and floating cells were then lysed and centrifuged and cytoplasmic fractions containing fragmented DNA were transferred to streptavidin-coated microtiter plates and incubated for 2 hours at room temperature with a mixture of anti–histone-biotin and anti–DNA-peroxidase antibodies. Quantitative determination of the amount of nucleosomes by the peroxidase retained in the immunocomplex was determined photometrically with 2,2′-azino-di-[3-ethylbenz-thiazoline-sulfonate-6-diammonium salt] as peroxidase substrate. DNA fragmentation in control and treated cells was expressed as absorbance at 405 nm. The apoptosis was also determined by the evaluation of the sub-G_1_ population by propidium iodide staining followed by flow cytometry analysis (see cell cycle analysis description).

### XBP-1 RT-PCR splicing assay

Reverse transcription polymerase chain reaction (RT-PCR) analysis was performed as described elsewhere [34]. XBP-1 cleavage assay was performed as previously described [35]. Briefly, XBP-1 was amplified using the gene-specific oligonucleotide primers: hXBP1.3S, 5′-A AAC AGA GTA GCA GCT CAG ACT GC-3′ and mXBP1.12AS, 5′- TC CTT CTG GGT AGA CCT CTG GGA G -3′. The PCR reaction cycle included 2 minutes of denaturation at 94°C followed by 35 cycles of 94°C for 30 seconds, 60°C for 30 seconds, and 72°C for 30 seconds. To distinguish the unspliced (473 bp) from the spliced (450 bp) band the PCR products were separated on a 3% agarose gel and visualized by UV after ethidium bromide staining.

### Cell cycle analysis

For analysis of cell cycle distribution, cells (9×10^5^) were plated into 100-mm tissue culture dishes and at approximately 30% confluence treated with 5 µM 4-oxo-4-HPR and/or 100 µM vitamin C. At different time points after the treatment, floating and attached cells were collected and washed twice with PBS, fixed in ice cold 70% ethanol and stored at -20°C until use. Subsequently, cells were rinsed with PBS and incubated with PBS containing 20 µg/mL propidium iodide (Sigma) and 1 mg/mL RNase A (Sigma). Cell cycle analysis was done using FACScan flow cytometer (Becton Dickinson, San Jose, CA). The percentage of cells in different phases of cell cycle was determined by ModFit LT cell cycle analysis software (Verity Software House, Topsham, ME), considering only cells with DNA content ≥2n. Apoptotic cells were identified as a sub-G_1_ population (DNA content <2n) and the percentage of cells in this pre-phase was calculated considering the totality of the events.

### Immunofluorescence Analysis

Cells, grown on glass coverslips slides in 24 mm Petri dishes, were fixed in 100% methanol at –20°C for 7 minutes, washed with PBS and then blocked at room temperature for 1 hour in 3% BSA/0.1% (v/v) Triton X-100/PBS. Cells were incubated overnight at 4°C in primary antibody, washed three times with PBS, and then incubated for another hour at room temperature with secondary antibody, washed with PBS and stained with Hoechst 33342 (Sigma) 2 µg/ml in PBS for 2 minutes. Slides were mounted with 0.1% (v/v) Mowiol (Calbiochem, San Diego, CA, USA) and viewed with a fluorescence microscope [images were recorded with a Spot Insight digital camera (Delta Sistemi) equipped with a system of image analysis (IAS 2000; Delta Sistemi)]. The following antibody was used: mouse anti-α-tubulin (Sigma). The secondary antibody used was anti-mouse Alexa 488 (Molecular Probes).

### Statistical analysis

Experiments and in vitro assays were carried out at least in triplicate. Differences between mean values were assessed by Student's t-test with two-sided P values <0.05 considered as statistically significant.

## Supporting Information

Figure S1 Effects of 4-oxo-4-HPR and vitamin C treatments on the expression of Bcl-2 family members. A2780 cells treated for 24 hours with 5 µM 4-oxo-4-HPR, with or without 100 µM vitamin C, were subjected to western blot analysis for the expression of Mcl-1, Bcl-2 and Bax. As a control for loading, the blots were incubated with actin antibody.
(0.51 MB TIF)

Figure S2 Effects of 4-oxo-4-HPR and vitamin C treatments on G2-M cell accumulation. Flow cytometric analysis of propidium iodide-stained A2780, T47D, HeLa and SK-N-BE cells treated for 24 hours with 5 µM 4-oxo-4-HPR with or without 100 µM vitamin C. Histograms show the percentage of cells in G2-M phase, according to the analysis performed with ModFit LT software. Data are means of three independent experiments; vertical bars are standard deviations. Asterisk indicates significant difference (P<0.05).
(0.47 MB TIF)
