# Supplementary figures and images for: 4-oxo-N-(4-hydroxyphenyl)retinamide: Two Independent Ways to Kill Cancer Cells
Source: PLoS One. 2010 Oct 14;5(10):e13362. doi: 10.1371/journal.pone.0013362 (PMC2954786; doi:10.1371/journal.pone.0013362)

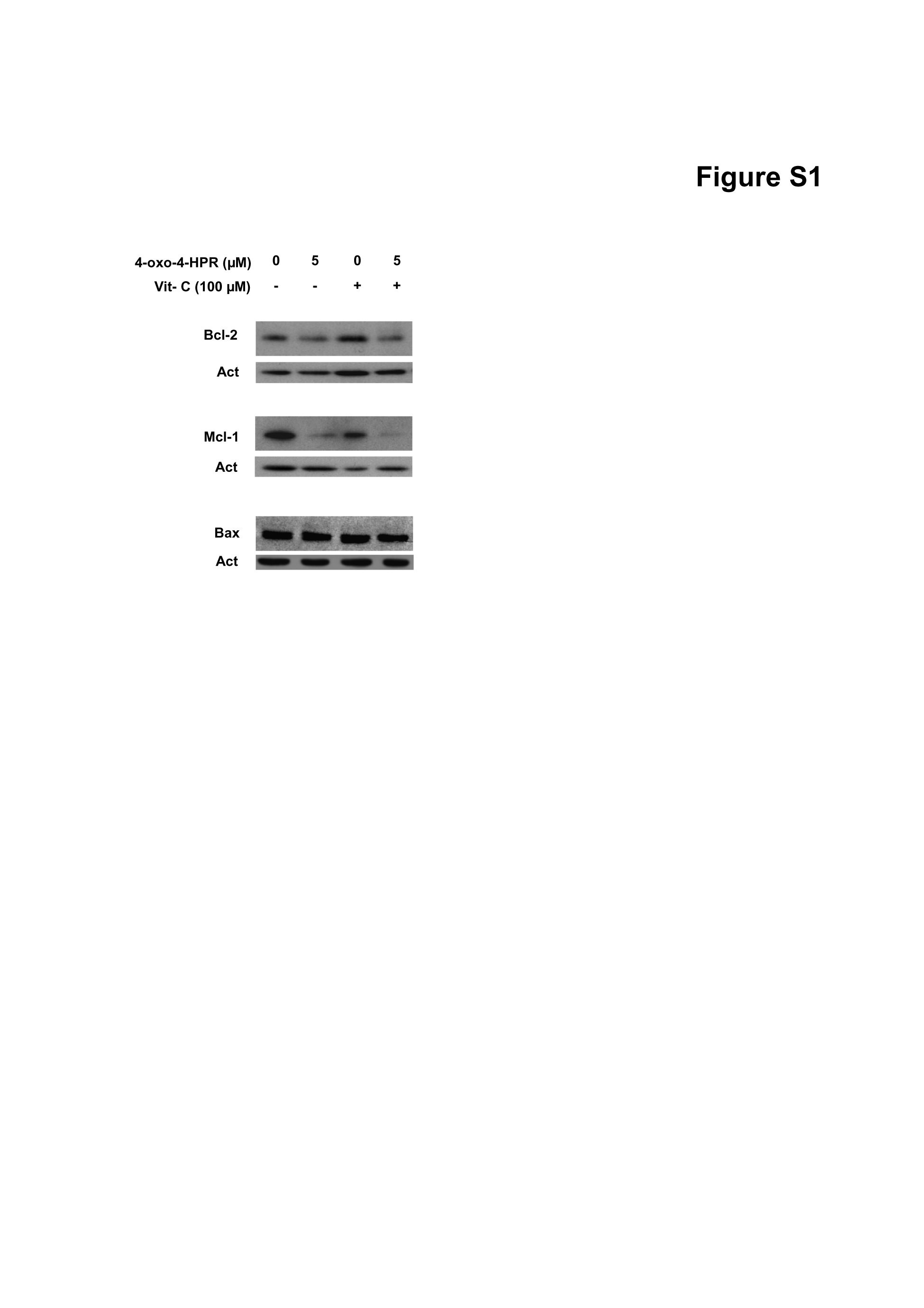

Supplement: Figure S1 — Effects of 4-oxo-4-HPR and vitamin C treatments on the expression of Bcl-2 family members. A2780 cells treated for 24 hours with 5 µM 4-oxo-4-HPR, with or without 100 µM vitamin C, were subjected to western blot analysis for the expression of Mcl-1, Bcl-2 and Bax. As a control for loading, the blots were incubated with actin antibody. (0.51 MB TIF) [file pone.0013362.s001.tif]

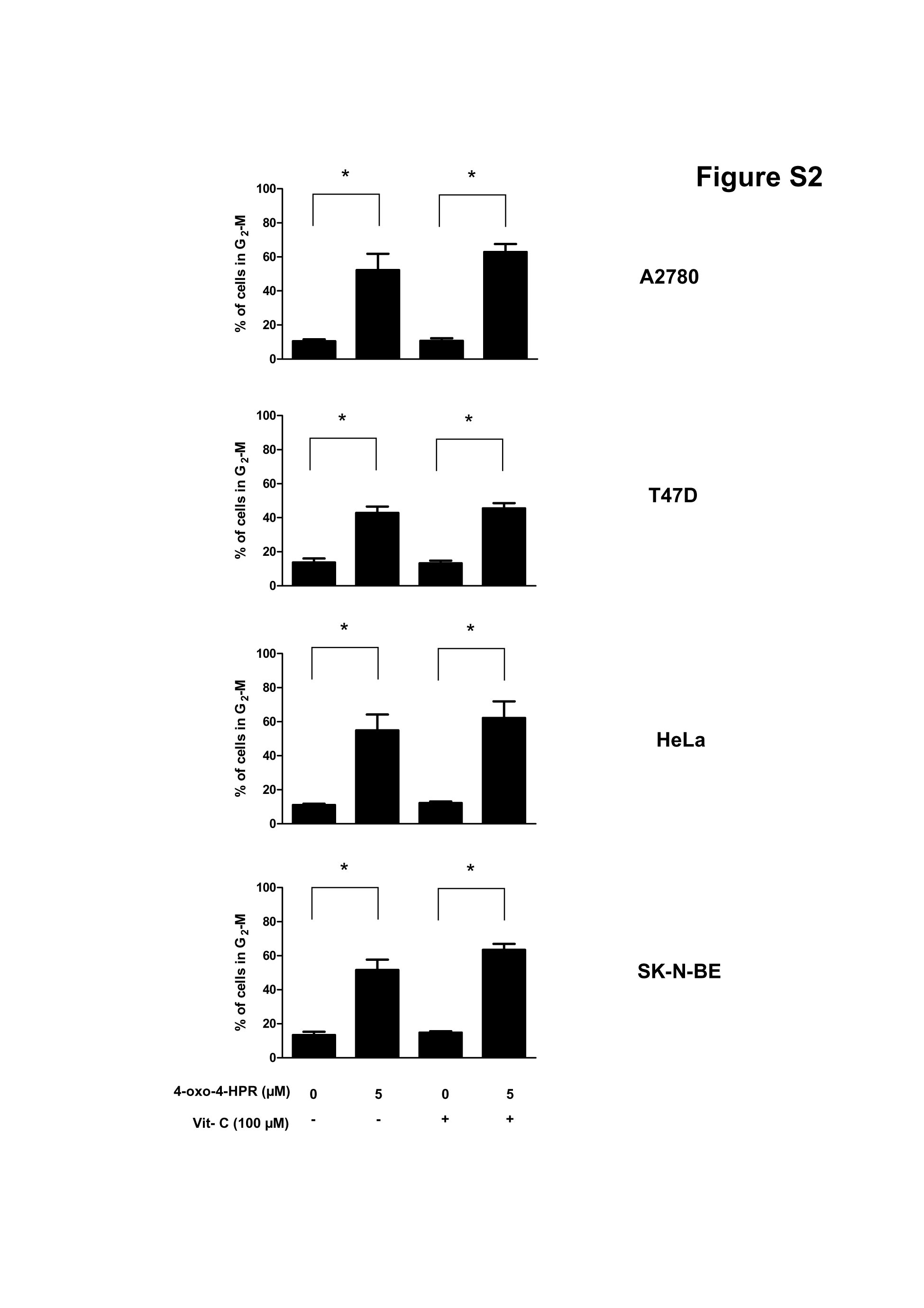

Supplement: Figure S2 — Effects of 4-oxo-4-HPR and vitamin C treatments on G2-M cell accumulation. Flow cytometric analysis of propidium iodide-stained A2780, T47D, HeLa and SK-N-BE cells treated for 24 hours with 5 µM 4-oxo-4-HPR with or without 100 µM vitamin C. Histograms show the percentage of cells in G2-M phase, according to the analysis performed with ModFit LT software. Data are means of three independent experiments; vertical bars are standard deviations. Asterisk indicates significant difference (P<0.05). (0.47 MB TIF) [file pone.0013362.s002.tif]
